# General Report & Recommendations in Predictive, Preventive and Personalised Medicine 2012: White Paper of the European Association for Predictive, Preventive and Personalised Medicine

**DOI:** 10.1186/1878-5085-3-14

**Published:** 2012-11-01

**Authors:** Olga Golubnitschaja, Vincenzo Costigliola

**Affiliations:** 1 Department of Radiology, Rheinische Friedrich-Wilhelms-University of Bonn, Sigmund-Freud-Str. 25, D-53105 Bonn Germany; 2 European Medical Association, Brussels, Belgium; 3 EPMA, Brussels, Belgium

**Keywords:** predictive, preventive and personalised medicine, integrative medicine, common/rare disease, diabetes/cancer/cardiovascular/neurodegenerative diseases, co-morbidities, well-being concept, research, education, healthcare, ethics, economy

## Abstract

This report is the collective product of word-leading experts working in the branches of integrative medicine by predictive, preventive and personalised medicine (PPPM) under the coordination of the European Association for Predictive, Preventive and Personalised Medicine. The general report has been prepared as the consortium document proposed at the EPMA World Congress 2011 which took place in Bonn, Germany. This forum analyzed the overall deficits and trends relevant for the top-science and daily practice in PPPM focused on the patient. Follow-up consultations resulted in a package of recommendations for consideration by research units, educators, healthcare industry, policy-makers, and funding bodies to cover the current knowledge deficit in the field and to introduce integrative approaches for advanced diagnostics, targeted prevention, treatments tailored to the person and cost-effective healthcare.

## Main Text

The full report can be viewed as PDF here http://www.epmajournal.com/content/pdf/1878-5085-3-14.pdf

